# Cervical Cancer Screening Services at Tertiary Healthcare Facility: An Alternative Approach

**DOI:** 10.31557/APJCP.2019.20.4.1265

**Published:** 2019

**Authors:** Kavitha Dhanasekaran, Chandresh Verma, Vipin Kumar, Roopa Hariprasad, Ruchika Gupta, Sanjay Gupta, Ravi Mehrotra

**Affiliations:** *ICMR-National Institute of Cancer Prevention and Research, Noida, UP, India. *

**Keywords:** Cervical cancer screening, PAP smear, opportunistic screening, population, based screening, LMIC

## Abstract

**Introduction:**

India fights massive cervical cancer burden. This article highlights an innovative feasible approach enabling tertiary hospitals to contribute to cancer prevention without compromising their primary mandate to provide treatment.

**Methodology:**

Since 1979, National Institute of Cancer Prevention and Research (NICPR) support a tertiary hospital in cervical cancer screening through a satellite clinic. Record review of 5328 attendees of this clinic between January-December 2016 was done. Pap-smear testing and reporting were performed by trained NICPR personnel. Patients’ demographics, reproductive history, Pap-test date, cytology results were recorded and results were communicated to respective units for further management.

**Results:**

Among 5328 women screened, 2% (96/5328) had abnormal cytology, which included malignancy(33%; 32/96), Atypical Squamous Cells-Undetermined Significance(ASC-US) (20%; 19/96), Atypical Glandular Cells(AGC) (23%; 22/96) with complaints of pain in lower abdomen 65.6%(59/90), white discharge per vaginum 46.7%(42/90) and backache 23.3%(21/90). In which, Muslims- 67% (65/96), illiterates- 58% (56/96). Age>35(p<0.001), parity>3(p<0.05), illiteracy (p<0.05), Muslim women (p<0.05) had positive association with abnormal cytology.

**Conclusion:**

Awareness about cervical cancer screening is the immediate need in resource-limited countries. Government hospitals in such countries should house dedicated preventive oncology unit for cancer screening.

## Introduction

Western countries have won their battle against cervical cancer to a large extent by adopting organized routine screening programs for cervical cancer (Hakama, 1982; Laara et al., 1987; Sasieni et al., 1995; Peto et al., 2004). India still accounts for one-quarter of the world’s cervical cancer burden (Ferlay et al., 2013). This is a leading public health concern.

In 2010, World Health Organisation(WHO) reported that India had the highest age-standardized incidence of cervical cancer in South Asia at 22, compared to 19.2 in Bangladesh, 13 in Sri Lanka (ICO 2014; WHO 2015; Sreedevi et al., 2015). The same year, government of India had taken initiative to reduce the incidence of cancers through prevention strategies and to improve the quality of life of those affected by cancer, including cervical cancer. A comprehensive program for control and prevention of cancers and non-communicable diseases like diabetes and cardiovascular diseases was framed in the name of National Programme for Prevention and Control of Cancer, Diabetes, Cardiovascular Disease and Stroke (NPCDCS), which emphasized on opportunistic screening, screening in camps for cervical cancer among women in rural areas and urban slums, and creating awareness among them (NPCDCS 2013).

The national cancer registries show a declining trend in cervical cancer in almost all parts of India. The annual decline percentage ranges from minimum of 1.3% in Bhopal to maximum 3.5% in Chennai (Nandakumar et al., 2013). Though the reasons for this could be multi-factorial like improved hygiene and socioeconomic status, the role of screening attributing to this declining trend is undeniable. Over the years, the incidence of the disease has come down with just opportunistic screening, and it can be further contained with adoption of an organized population-based screening program.

Systematic population-based screening for cervical cancer using conventional Papanicolaou (Pap) smear has played an inevitable role in the decline of incidence of cervical cancer, mortality, and morbidity caused by the same (Fidler et al 1968; Hakama et al., 1976; Hakama 1982; Laara et al., 1987; Lynge et al., 2000). Though opportunistic screening is less effective than systematic screening, it has proved to have a significant impact on reducing the incidence of cervical cancer in some western countries (Magnus et al., 1986; Lynge et al., 1989; Nieminen et al., 1999). The combination of an organized and opportunistic screening program for cervical cancer has proved to be more effective than any single method.

Thanks to modern medical science, over the decades, many methods have been suggested as preferred modality for cervical cancer screening according to the resources available. Method used for screening could be adopted from the national guidelines for population-based screening for cervical cancer among women of age group 30-65 years along with other common cancers (MoHFW 2016) as per the available resources at the health facility. Screening modalities may differ, but screening women at least once in their lifetime are likely to substantially bring down the disease burden in our country.

National Institute of Cancer Prevention and Research (formerly known as Institute of Cytology and Preventive Oncology [ICPO]) was functioning in Maulana Azad Medical College (MAMC) campus from 1979 till 2005. The Institute’s mandate being cancer prevention, it provided cytology-based cervical cancer screening services to collaborating hospitals. NICPR had set up a cervical cancer screening clinic run by paramedical staff, at Lok Nayak Jai Prakash Narayan (LNJP) hospital associated with MAMC, a huge tertiary health care facility under the government of Delhi, which caters to people from Delhi and neighboring states like Haryana and Uttar Pradesh. Since then, even after change of its location to Noida, it has been extending its support for cervical cancer screening, with a service motive, by providing Pap smear services.

Tertiary healthcare facilities are mostly overcrowded because of the availability of advanced medical facilities under one roof. The clinicians and pathologists are often overburdened. Cancer screening in such circumstances takes a back seat. There are simple screening tools like visual inspection of cervix with 5% acetic acid (VIA) or Pap smear that could be performed at ease. The screen-positive women can be subsequently referred for colposcopic examination and further management within the same facility. The WHO recommended “Screen and Treat” method can be easily adopted to address the loss to follow-up (LTFU) challenge, often encountered in these settings.

In 2016, when the Operational Framework for Management of Common Cancers was released by government of India, we realized the need to disseminate the results more effectively. We electronically entered the data of women who attended our extension clinic from January-December 2016. We performed retrospective analysis of this data, with the objective of studying the association between socio-demographics and abnormal cytology results on Pap test in the population screened and to assess the usefulness of the model satellite cervical cancer screening clinic run by paramedical staff.

The result from this retrospective analysis may motivate others to take their first step forward towards reducing the burden of this disease in our nation. It may also induce health care professionals to implement screening for cervical cancer at their facilities. Our initiative could be just a drop in an ocean. Indeed! Drops make the ocean.

## Materials and Methods

*Methodology*

Retrospective record review was performed for total of 5,328 women who were referred from department of Gynecology at LNJP hospital for Pap smear to our satellite cervical cancer screening clinic between January-December 2016. Pap smear test was collected by trained medical social workers from NICPR using Ayre’s spatula and endocervical brush. The slides were reported by experienced cytopathologists from NICPR following the latest Bethesda system of reporting (TBS 2001). Data on age, marital status of the patient, reproductive history, occupation of their spouses and their current gynecological symptoms (if any), date of Pap smear performed and the result were retrieved from the pre-filled proforma. All the Pap smear results were communicated to the respective referral units. After screening, all screened women were directed to their concerned referral units to collect their reports, to ensure that those with abnormal cytology were subjected to further necessary evaluations and treated appropriately.

The data analysis was carried out in SPSS 21.0. For the quantitative data; descriptive statistics (Mean, SD) were calculated and independent student t-test was employed to evaluate the significant mean difference between the two groups. For the qualitative data, percentages were calculated using Chi-square and Fisher’s exact test, whichever was applicable. 

## Results

Among 5328 women screened for cervical cancer using cytology, 2% (96) had abnormal results. Among women with abnormal cytology (96/5,328), the most frequent abnormality encountered included malignancy in 33% (32/96), ASC-US 20% (19/96) and AGC 23% (22/96). High grade squamous intraepithelial lesion (HSIL) and Low grade squamous intraepithelial lesion (LSIL) comprised 9% (9/96) and 7% (7/96) respectively, of total epithelial cell abnormalities. The chief complaints among women with abnormal cytology were pain in the lower abdomen (61.5%), white discharge per vaginum (43.8%) and backache (21.9%). Among women with abnormal cytology (96/5,328), mean age was 45 years, 65% (62/96) were multiparous women with >3 children and 58% (56/96) women were illiterate. Sixty-seven percent (65/96) of them followed Islam religion. About 6% (6/96) of women with abnormal Pap smear results were asymptomatic. The socio-demographic details of the women screened the frequency of cytological abnormalities and the most common complaints have been enlisted in Tables 1, 2 and Table 3 respectively.

## Discussion

From the time of its launch, the extended satellite clinic was functioning only as a patient care facility. Hence, no comprehensive data capture was performed in previous years and data available was not transcribed in electronic form. Entering data from past 40 years in digital format was not a feasible option. This factor became a serious limitation in disseminating the full benefits of having this model extended cervical cancer screening facility in a tertiary hospital setting which has saved hundreds of women from the killer disease.

**Table 1:** Demographic Profile of Women Screened (n=5328)

Variable	Categories	Normal n (%)	Abnormal n (%)	p-value Chi-square test	CI (95%) Min-Max
Age	< 35	2,838 (54.2)	30 (31.3)		1.599 – 2.373
	> 35	2,394 (45.8)	66 (68.8)	p<0.001	
Age at Marriage	< 18	1,856 (35.5)	39 (40.6)	NS	0.533 – 1.212
	>18	3,376(64.5)	57 (59.4)		
Parity	< 3	2,770(52.9)	34 (35.4)		1.346 – 3.128
	>3	2,462 (47.1)	62 (64.6)	p<0.05	
Number of Abortion	<4	5,154 (98.5)	95 (99.0)	NS	0.096 – 5.052
	>4	78 (1.5)	1 (1.0)		
Education	Other	2,905 (55.5)	40 (41.7)		1.161 – 2.632
	Illiterate	2,327 (44.5)	56 (58.3)	p<0.05	
Religion	Muslim	2,900 (55.4)	65 (67.7)	p<0.05	0.385 – 0.913
	Other	2,332 (44.6)	31 (32.3)		
Husband Occupation	Laborer/ driver	1,000 (19.1)	14 (14.6)	NS	0.782 – 2.450
	Other	4,232 (80.9)	82 (85.4)		

**Table 2:** Abnormal Cytology Results (n = 96)

Cytology Result	Abnormal n (%)
Malignant	32 (33.3)
HSIL	9 (9.4)
LSIL	7 (7.3)
ASC-H	5 (5.2)
AGC	22 (22.9)
ASC-US	19 (19.8)
Atypical cells seen	2 (2.1)

HSIL, High-grade squamous intraepithelial lesion; LSIL, Low-grade squamous intraepithelial lesion; ASC-H, Atypical squamous cells- cannot exclude high-grade squamous intraepithelial lesion; AGC, Atypical glandular cells; ASC-US, Atypical squamous cells- undetermined significance

**Table 3:** Common Complaints among Screened Positives (n=90)

Complaints*	Abnormal n (%)	P-value
White discharge	42 (46.7)	
Pain lower abdomen	59 (65.6)	P<0.001
Postmenopausal bleeding	14 (15.6)	
Backache	21 (23.3)	
Contact bleeding	2 (2.2)	
Dysuria	2 (2.2)	
Itching	3 (3.3)	
Menorrhagia	6 (6.7)	
Prolapse	5 (5.6)	
Burning micturition	2 (2.2)	
Dyspareunia	0 (0.0)	
Irregular periods	4 (4.4)	
No Complaints	6 (6.3)	

This review of retrospective one-year (2016) data shows that multiparous women with >3 issues had significantly higher risk of cervical abnormalities (P=0.001, 95% C.I [1.346-3.128]). Illiterate women also had increased risk of cervical abnormalities compared to educated women (P=0.007, 95% C.I [1.161-2.632]) and women from Islam religion had more risk of cervical abnormality (P=0.016, 95% C.I [0.385-0.913]). In contrary to the past literature, in our study, Muslim women had higher incidence of cervical cancer compared to those from other religions. This review highlights few other important factors like illiteracy, which can be a surrogate for lack of awareness about cervical cancer and its preventive measures as one important reason for increased cervical abnormality among these women who utilized the cancer screening services at the model clinic. The other factor, which without denial catches our attention, is the fact that 6% of women with cytological abnormality were asymptomatic. This forms a relevant ground to promote and scale up systematic cervical cancer screening in our population. The most common abnormality encountered being malignancy, showcases the fact that along with opportunistic screening, where only symptomatic women are screened, population-based screening should go hand in hand. This will ensure early detection of precancerous lesions rather than they being diagnosed in the advanced stages.

Operational Framework for Management of Common Cancers offers a feasible working model for population-based screening of the common cancers, wherein the paramedical workforce is suggested to do the primary level of cancer screening. This showcases that, if paramedical workforce can be appropriately trained and utilized, there is a possibility of screening 80% of eligible population within a three-year span. With a slight shift in focus from doctors to paramedical personnel as the frontline cancer screening workforce, the huge impending pressure on specialists at tertiary care centers is likely to reduce substantially. The expertise of medical doctors may rather be utilized in compelling treatment priorities.

The model satellite cervical cancer screening clinic, run by NICPR at the tertiary care hospital is an illustrative model of the same concept that the government of India is offering the nation to adopt for population-based cancer screening program. Four decades of successful functioning of this clinic has generated sufficient evidence that this model is a feasible and innovative approach for cancer screening. NICPR and this particular tertiary care hospital collaboratively divided their responsibilities and functioned efficiently to meet their mandates. This clinic provides cervical cancer screening services by cytology to those referred by specialists from gynecology department of the tertiary care hospital. At the clinic, trained paramedical staffs provide cervical cancer screening services by performing Pap test for the patients. By directing the patients to the model clinic, the specialists are able to cater to more patients with treatment priorities and the workload of a specialist is distributed to allied medical personnel. Since the patients with abnormal cytology results are usually much less in number compared to those subjected to screening, the specialists need to provide expert services only to that small group of patients who need further evaluation and management.

Most of the Low-Income Countries (LICs), and Lower-Middle Income Countries (LMICs) face similar challenges like overcrowded tertiary hospitals and huge cancer burden in health systems like ours. Cervical cancer prevention and control are major public health concern. Just like India, other countries like Bangladesh, Nepal, and a few Asian and African countries are also at war against cervical cancer. Unfortunately, in most LICs and LMICs infectious diseases are rampant and major sector of health system’s workforce is directed towards treating these while screening for cancers takes a back seat.

*Measures to overcome the limitations of cervical screening in an overburdened health system in India and other LICs and LMICs*

Instead of using cytology, with its inherent limitations, as a screening tool, visual inspection of cervix with 5% acetic acid (VIA), the recommended screening tool for LICs and LMICs, can be adopted relatively easily. This could help reduce the burden for pathologists in tertiary hospitals. Moreover, VIA is a simple test which any trained paramedical staff can perform even with limited training and resources. “See and Treat” approach, WHO’s most recommended treatment method to avoid LTFU of patients, can be practiced in these settings. Medical and paramedical personnel can do reasonably good screening job with minimal training. Other possible suggestion would be for tertiary hospitals to tie up with other institutions/facilities for preventive oncology services. Our concept of an extended model cancer screening unit is a good example and proof of a feasible working system in this regard. The most effective way is to run a dedicated Preventive Oncology Unit (POU) utilizing the paramedical workforce, which is the greatest strength of health systems in India and other LICs and LMICs. This unit must focus on cancer screening for all common cancers in the country viz cervical, breast and oral cancer in India. This comprehensive approach is likely to be more efficient and productive, rather than just screening for cervical cancer. The POU could recruit trained paramedical staff to perform the primary level screening for common cancers and only a limited number of medical personnel to monitor them and to refer the patients with positive test results to the concerned departments for further evaluation and treatment. If practiced efficiently by healthcare facilities at all levels, this approach could substantially cut down the workload not just for the clinicians and pathologists in tertiary healthcare facilities but also go a long way in reducing the cancer burden faced by the country.

In addition, there is a dire need to scale-up the implementation of cervical cancer screening programs in all government health care facilities, at all the levels, inclusive of primary health care centers, medical college hospitals, and district hospitals including counseling facilities for women to minimize high-risk behaviors.

## Limitations

Since LNJP hospital caters medical care for patients from Delhi and its neighboring states like Haryana and Uttar Pradesh, the attendees of our model clinic were largely a migrating population. LTFU was the major challenge that was difficult to address. Since the patients were referred to the clinic from different units, keeping track of those with positive Pap smear results and those who underwent further evaluation and treatment in their respective units of the gynecology department was a demanding task.

In conclusion, the extended cervical cancer screening clinic run by NICPR, where the paramedical staff performs the screening at LNJP, has aided in detecting premalignant and malignant cervical pathology through cytology-based screening. These women are subsequently managed according to standard protocol. This is a working model for cancer screening that could be adopted all over our country and also replicated in other low resource settings which share similar health system concerns. Country-wide awareness campaigns about cervical cancer screening and motivating women to undergo routine cervical cancer screening are the most essential elements which are immediately needed to catalyze cancer prevention in our country. Diagnosing and treating cervical cancer at the advanced stage is a great financial burden on the government. Prevention is a more feasible strategy. Rather than investigating and treating only symptomatic patients, all government hospitals including medical college hospitals and district hospitals must start a dedicated POU and should initiate cervical cancer screening as a routine, if elimination of cervical cancer is our pivotal goal. Adding oral and breast cancer screening components in the existing cervical cancer screening system will make it more comprehensive and a win-win situation for both the nation’s health system and the participants.

“*No More Deaths from Preventable Cancers*” must be our motto.

## Notes

Compliance with ethical standards.

## Ethical approval

 All procedures performed in this study involving human participants were in accordance with the ethical standards of the institutional and/or national research committee and with the 1964 Helsinki declaration and its later amendments or comparable ethical standards. For this type of study formal consent is not required.

## Conflict of Interest

The authors declare that they have no conflict of interest.
